# Editorial for Special Issue “Plant Mitochondria”

**DOI:** 10.3390/ijms19123849

**Published:** 2018-12-03

**Authors:** Nicolas L. Taylor

**Affiliations:** ARC Centre of Excellence in Plant Energy Biology, School of Molecular Sciences and Institute of Agriculture, The University of Western Australia, Crawley, WA 6009, Australia; nicolas.taylor@uwa.edu.au; Tel.: +61-8-6488-1107; Fax: +61-8-6488-4401

The primary function of mitochondria is respiration, where catabolism of substrates is coupled to adenosine triphosphate (ATP) synthesis via oxidative phosphorylation (OxPhos). Organic acids such as pyruvate and malate produced in the cytosol are oxidised in mitochondria by the tricarboxylic acid (TCA) cycle and subsequently by the electron transport chain (ETC). Energy released by this oxidation is used to synthesise ATP, which is then exported to the cytosol for use in biosynthesis and growth. In plants, mitochondrial composition is relatively complex and flexible and has specific pathways to enable continuous survival during abiotic stress exposure and to support photosynthetic processes in illuminated leaves.

Plant mitochondria are double-membrane organelles where the inner membrane is invaginated to form folds known as cristae to increase the surface area of the membrane. The outer membrane contains relatively few proteins (<100) and is permeable to most small compounds (<Mr = 5 kDa) due to the presence of the pore-forming protein VDAC (voltage dependent anion channel), which is a member of the porin family of ion channels. The inner membrane is the main permeability barrier of the organelle and controls the movement of molecules by means of a series of carrier proteins, many of which are members of mitochondrial substrate carrier family (MSCF). The inner membrane also houses the large complexes that carry out electron transfer in two inter-connected pathways that finish with two terminal oxidases. It is also the site of oxidative phosphorylation (OxPhos) and contains a non-phosphorylating bypass of the classical ETC. The inner membrane also encloses the soluble matrix which contains the enzymes of the TCA cycle and many other soluble proteins involved in a myriad of mitochondrial functions.

Mitochondria are semi-autonomous organelles with their own DNA, protein synthesis, and degradation machinery. The proteins encoded by the mitochondrial genome undergo a range of post-transcriptional and post-translational processing during their synthesis. The mitochondrial genome also encodes a number of pollen abortion related genes involved in controlling plant fertility in a process known as cytoplasmic male sterility (CMS). These CMS plants are used to produce hybrids that benefit from hybrid vigor or heterosis, producing greater biomass and yield. However, the mitochondrial genome encodes only a small portion of the proteins which make up the mitochondrion; the rest are encoded by nuclear genes and synthesised in the cytosol. These proteins are then transported into the mitochondrion by the protein import machinery and assembled with the mitochondrially synthesised subunits to form the large respiratory complexes and other proteins.

Stress tolerance is a very complex trait, involving a multitude of developmental, physiological, and biochemical processes. Compared to other organelles, plant mitochondria are disproportionately involved in stress tolerance, probably because they are a convergence point between metabolism, signaling, and cell fate [1]. Mitochondria are also the site of production of reactive oxygen species (ROS), with the ubiquinone pool and components in Complex I and Complex III the main sites of production. Recently, Complex II has also been shown to produce significant superoxide [2]. Under normal steady state conditions, ROS production is controlled by a complex array of antioxidant enzymes and small molecules that scavenge ROS and limit mitochondrial and cellular damage. However, under some conditions these defences can become overwhelmed and ROS accumulate, leading to damage of proteins, lipids, and DNA. 

The number of mitochondria per cell varies with tissue type, with more active cells with high energy demands, such as those in growing meristems, generally equipped with larger numbers of mitochondria per unit cell volume and typically these show faster respiration rates. Research on plant mitochondria has rapidly developed in the last few decades with the availability of genome sequences for a wide range of model and crop plants. Recent prominent themes in the plant mitochondrial research include linking mitochondrial composition to environmental stress responses and how this oxidative stress impacts upon mitochondrial function. Similarly, interest in the signaling capacity of mitochondria (the role reactive oxygen species, retrograde, and anterograde signaling) has revealed the transcriptional changes of stress responsive genes as a framework to define specific signals emanating to and from the mitochondrion. There has also been considerable interest in RNA metabolic processes in plant mitochondria including RNA transcription, RNA editing, the splicing of group I and group II introns, and RNA degradation and translation. Despite their identification more than 100 years ago plant mitochondria remain a significant area of research in the plant sciences. 

In this Special Issue, “Plant Mitochondria”, a total of 19 articles were accepted with 15 original research articles and 4 review articles broadly covering the field of plant mitochondrial research (Table 1). Manuscripts focused on protein synthesis and degradation [3,4,5,6], abiotic stress [7,8,9,10], OxPhos [11,12,13,14], protein import [15,16,17], ROS and antioxidants [18], and CMS [19,20]. 

A number of research articles in this Special Issue focused on the responses of mitochondria to abiotic stress, with studies that examined thermal stress (both hot and cold), salinity, and drought. Arimura et al. [7] demonstrated that cold induced mitochondrial fission (which was previously thought to involve the action of both a dynamin-related protein) DRP3A and another plant specific factor ELM1, only requires DRP3A in Arabidopsis. At the same time, they showed that an *ELM1* paralogue (*ELM2*) seemed to have only a limited role in mitochondrial fission in an *elm1* mutant, suggesting that Arabidopsis has a unique, cold induced mitochondrial fission that involves only DRP3A to control the size and shape of mitochondria. The mitochondrial transcription termination factors (mTERFs) which are involved in the control of organellar gene expression (OGE) with mutations in some characterized mTERFs (resulting in plants that have altered responses to salt, high light, heat, or osmotic stress) suggesting a role for these proteins in abiotic stress tolerance. Here Robles et al. [8] showed that strong loss of function mutant *mterf6-2* was hypersensitive to NaCl and mannitol during seedling establishment, while *mterf6-5* showed a greater sensitivity to heat later in development. Rurek et al. presented a pair of research papers that used physiological, proteomic, and transcript analysis approaches to examine the thermal (hot and cold) and drought responses of cauliflower mitochondria [9,10]. In the thermal studies they identified a number of proteins that were temperature responsive including components of OxPhos, photorespiration, porin isoforms, and the TCA cycle. Similarly, in the drought analysis, which examines three different cauliflower cultivars, both OxPhos components and porin isoforms were seen to change in abundance, indicating a significant differential impact on mitochondrial biogenesis between the three cultivars, giving us new insights into the abiotic stress responses of the *Brassica* genus.

Male sterility refers to the inability of a plant to make viable pollen. It can be mediated through nuclear genes leading to genic male sterility (GMS) or through mitochondrial proteins interacting with nuclear genes, leading to cytoplasmic male sterility (CMS). Both GMS and CMS are widely used in agricultural production for the production of hybrid crops that benefit from heterosis. In this Special Issue Štorchová [20] presents a comprehensive review of the role of non-coding RNA in the CMS of flowering plants, while Reddemann and Horn [19] presented research examining the role of *atp9* in the male sterility of CMS PET2 in sunflower. Here they showed that CMS PET2, which has the potential to become an alternative CMS source for commercial breeding, has a duplicated *atp9* with a 271-bp-insertion in the 5’ region of one of the *atp9* genes which results in two unique open reading frames (*orf288* and *orf231*). The reduced anther-specific co-transcription of these open reading frames in fertility-restored hybrids supports their involvement in male sterility in CMS PET2.

A total of five papers we submitted examining OxPhos, with two of these focused on identifying non-phosphorylating bypasses of the classical ETC. Wanniarachchi et al. [14] identified and characterised the alternative oxidase (AOX) and the type II NAD(P)H dehydrogenases (NDs) of rice and barley, while Velada et al. [13] characterized the AOX1 subfamily in *Olea europaea* cv. Galega Vulgar (European olive). Podgórska et al. [12] examined the Complex 1 mutant *fro1* (*frostbite 1*) that has a point mutation in the 8 kDa Fe-S subunit NDUFS4 grown on different nitrogen sources. When these plants were grown on NO_3_^-^ they showed a carbon flux towards nitrogen assimilation and energy production, whereas cellulose integration into the cell wall was restricted. In contrast they showed improved growth on NH_4_^+^ and not the expected ammonium toxicity syndrome. Similarly, Podgórska et al. [11] showed that plants with external NADPH-dehydrogenase (NDB1) knockdown were resistant to NH_4_^+^ treatment and had milder oxidative stress symptoms with lower ROS accumulation and induction of glutathione peroxidase-like enzymes and peroxiredoxins antoxidants. Mansilla et al. provided a comprehensive review of the composition and biogenesis of the terminal oxygen acceptor cytochome *c* oxidase (Complex IV) in yeast, mammals, and plants. This revealed that while plants retain many biogenesis features common to other organisms, they have also developed plant specific features.

As the majority of proteins that function in mitochondria are imported from nuclear encoded cytosolic synthesized proteins, studies understanding the process of how mitochondrial protein import is controlled and regulated is vital to alter mitochondrial functions. Here Zhao et al. [17] examined the intergenomic transfer (IGT) from a broad evolutionary perspective by accessing data from nuclear, mitochondrial, and chloroplast genomes in 24 plants, and showed that mitochondrial transfer occurs in all plants examined. Additionally, Avelange-Macherel et al. [15] used two paralogues of late embryogenesis abundant proteins (LEA) (LEA38 (mitochondrial) and LEA2 (cytosolic)) to examine the influence of amino acid sequence of mitochondrial targeting sequences (MTS) on subcellular localisation. They showed that by combining substitution, charge invasion, and segment replacement, they were able to redirect LEA2 to mitochondria, providing an explanation for the loss of mitochondrial localistion after duplication of the ancestral gene. Kolli et al. [16] provided a complete review of unique aspects of plant mitochondrial inner membrane protein insertion using Complex IV as a case study, which revealed the use of Tat machinery for membrane insertion of the Rieske Fe/S protein.

Two papers examined the mitochondrial protease FTSH4, one looking at the impact of a *ftsh4* mutant on meristem proliferation [3], and another identifying physiological substrates and interaction partners using a trapping approach and mass spectrometry [4]. Dolzblasz et al. showed that plants lacking AtFTSH4 show a cessation of growth at both the shoot and root apical meristems when grown at 30 °C, and that this arrest is caused by cell cycle dysregulation and the loss of cell identity. Opalińska et al. revealed a number of novel putative targets for FTSH4 including the mitochondrial pyruvate carrier 4 (MPC4), presequence translocase-associated motor 18 (PAM18), and succinate dehydrogenase (SDH) subunits. Additionally, they showed that FTSH4 is responsible for the degradation of oxidatively damaged proteins in mitochondria. Plant mitochondria contain numerous group II introns which reside in genes. Here Zmudjak et al. [6] showed that the nMAT2 maturase and the RNA helicase PMH2 associate with their intron-RNA targets in large ribonucleoprotein particle in vivo and the splicing efficiencies of the joint intron targets of nMAT2 and PMH2 are more strongly affected in a double *nmat2/pmh2* mutant-line. Together this suggests that these proteins serve as components of a proto-spliceosomal complex in plant mitochondria. Robles et al. [5] provides a thorough review of the phenotypic effects on plant development displayed by mutants of mitoribosomal proteins (mitoRPs) and how they contribute to the elucidation of plant mitoRPs function, the mechanisms that control organelle gene expression, and their contribution to plant growth and morphogenesis.

Mao et al. [18] examined the application of 0.05 mM NO in aged oat seeds and saw an improvement in seed vigor and increased H_2_O_2_ scavenging ability in mitochondria. Accompanying this were higher activities of CAT, GR, MDHAR, and DHAR in the AsA-GSH scavenging system, enhanced TCA cycle-related enzymes (malate dehydrogenase, succinate-CoA ligase, fumarate hydratase), and activated alternative pathways.

Overall, the 19 contributions published in this special issue illustrate the advances in the field of plant mitochondria and I look forward to catching up with the plant mitochondrial community at the next biannual meeting in Ein Gedi, Israel (https://www.icpmb2019.com/).

## Figures and Tables

**Table 1:** Contributors to the Special Issue “Plant Mitochondria”.

Authors	Title	Topics	Type
Arimura et al. [7]	Cold Treatment Induces Transient Mitochondrial Fragmentation in Arabidopsis thaliana in a Way that Requires DRP3A but not ELM1 or an ELM1-Like Homologue, ELM2	Abiotic stress	Original Research
Robles et al. [8]	The Characterization of Arabidopsis mterf6 Mutants Reveals a New Role for mTERF6 in Tolerance to Abiotic Stress	Abiotic stress	Original Research
Rurek et al. [9]	Cold and Heat Stress Diversely Alter Both Cauliflower Respiration and Distinct Mitochondrial Proteins Including OXPHOS Components and Matrix Enzymes	Abiotic stress	Original Research
Rurek et al. [10]	Mitochondrial Biogenesis in Diverse Cauliflower Cultivars under Mild and Severe Drought. Impaired Coordination of Selected Transcript and Proteomic Responses, and Regulation of Various Multifunctional Proteins	Abiotic stress	Original Research
Reddemann et al. [19]	Recombination Events Involving the atp9 Gene Are Associated with Male Sterility of CMS PET2 in Sunflower	Cymiddlelasmic Male Sterility	Original Research
Štorchová et al. [20]	The Role of Non-Coding RNAs in Cymiddlelasmic Male Sterility in Flowering Plants	Cymiddlelasmic Male Sterility	Review
Mansilla et al. [21]	The Complexity of Mitochondrial Complex IV: An Update of Cytochrome c Oxidase Biogenesis in Plants	Oxidative Phosphorylation	Review
Podgórska et al. [12]	Nitrogen Source Dependent Changes in Central Sugar Metabolism Maintain Cell Wall Assembly in Mitochondrial Complex I-Defective frostbite1 and Secondarily Affect Programmed Cell Death	OxPhos	Original Research
Velada et al. [13]	AOX1-Subfamily Gene Members in Olea europaea cv. “Galega Vulgar”—Gene Characterization and Expression of Transcripts during IBA-Induced In Vitro Adventitious Rooting	OxPhos	Original Research
Wanniarachchi et al. [14]	Alternative Respiratory Pathway Component Genes (AOX and ND) in Rice and Barley and Their Response to Stress	OxPhos	Original Research
Podgórska et al. [11]	Suppression of External NADPH Dehydrogenase—NDB1 in Arabidopsis thaliana Confers Improved Tolerance to Ammonium Toxicity via Efficient Glutathione/Redox Metabolism	OxPhos	Original Research
Avelange-Macherel et al. [15]	Decoding the Divergent Subcellular Location of Two Highly Similar Paralogous LEA Proteins	Protein Import	Original Research
Kolli et al. [16]	Plant Mitochondrial Inner Membrane Protein Insertion	Protein Import	Review
Zhao et al. [17]	The Roles of Mitochondrion in Intergenomic Gene Transfer in Plants: A Source and a Pool	Protein Import	Original Research
Dolzblasz et al. [3]	Impairment of Meristem Proliferation in Plants Lacking the Mitochondrial Protease AtFTSH4	Protein Synthesis and Degradation	Original Research
Opalińska et al. [4]	Identification of Physiological Substrates and Binding Partners of the Plant Mitochondrial Protease FTSH4 by the Trapping Approach	Protein Synthesis and Degradation	Original Research
Robles et al. [5]	Emerging Roles of Mitochondrial Ribosomal Proteins in Plant Development	Protein Synthesis and Degradation	Review
Zmudjak et al. [6]	Analysis of the Roles of the Arabidopsis nMAT2 and PMH2 Proteins Provided with New Insights into the Regulation of Group II Intron Splicing in Land-Plant Mitochondria	Protein Synthesis and Degradation	Original Research
Mao et al. [18]	Nitric Oxide Regulates Seedling Growth and Mitochondrial Responses in Aged Oat Seeds	ROS & Antioxidants	Original Research

## Abbreviations

AOX	Alternative Oxidase
CMS	Cytoplasmic Male Sterility
ETC	Electron Transfer Chain
GMS	Genic Male Sterility
IGT	InterGenomic Transfer
LEA	Late Embryogenesis Abundant proteins
mitoRPs	mitochondrial Ribosomal Proteins
MPC4	Mitochondrial Pyruvate Carrier 4
MSCF	Mitochondrial Substrate Carrier Family
mTERFs	mitochondrial Transcription TERmination Factors
NDs	Type II NAD(P)H dehydrogenases
NDB1	external NAD(P)H dehydrogenase
OGE	Organellar Gene Expression
OxPhos	Oxidative Phosphorylation
PAM18	Presequence translocase-Associated Motor 18
ROS	Reactive Oxygen Species
SDH	Succinate DeHydrogenase
TCA	Tricarboxylic Acid Cycle
VDAC	Voltage Dependent Anion Channel
